# Influence of the route of exposure and the matrix on the sensitisation potency of a major cows’ milk allergen

**DOI:** 10.1186/s13601-015-0047-x

**Published:** 2015-01-28

**Authors:** Sophie Wavrin, Herve Bernard, Jean-Michel Wal, Karine Adel-Patient

**Affiliations:** Unité INRA d’Immuno-Allergie Alimentaire, IBiTec-S – SPI, Bât. 136 – CEA de Saclay, 91191 Gif-sur-Yvette Cedex, France; AgroParisTech - Department SVS, 16 rue Claude Bernard, F-75231 Paris Cedex 05, France

**Keywords:** Food allergy, Cows’ milk, Mice, Cutaneous exposure, Respiratory exposure

## Abstract

**Background:**

Allergic sensitisation to food may occur through non-gastrointestinal routes such as via skin or lung. We recently demonstrated in mice that cutaneous or respiratory pre-exposures to peanut proteins on intact epithelia induce a Th2 priming and allow subsequent oral sensitization without the use of adjuvant. We then aimed to assess the impact of a similar pattern of exposure to another relevant food allergen, cows’ milk.

**Findings:**

The humoral and cellular immune response induced in BALB/cJ mice after repeated cutaneous applications on intact skin or after intranasal administration of cows’ milk proteins was analysed. In order to assess the potential effect of the food matrix, we used either a purified major cows’ milk allergen, β-lactoglobulin (BLG), or whole cows’ milk containing the same amount of BLG. We then studied the impact of these pre-exposures on a subsequent oral exposure to milk in the presence or absence of the mucosal Th2 adjuvant, Cholera toxin (CT). Cutaneous applications of milk induced production of BLG-specific IgE and IgG1 in 5 and 8 mice out of 20 respectively, whereas purified BLG alone did not. Intranasal exposure to milk, but not to BLG, led to BLG-specific IgG1 production in 8 out of 20 mice. Notably, cutaneous pre-exposure to milk favours further oral sensitisation without CT, while intra-nasal pre-exposure to BLG prevents further experimental sensitisation.

**Conclusions:**

Altogether, our results thus demonstrated that the immune response induced after non-gastrointestinal exposure to food depends on the allergen, the matrix and the route of exposure.

## Findings

### Introduction

Type I hypersensitivity immune responses represent about 60% of cases of cows’ milk allergy, the most common food allergy in early life. It involves the production of IgE antibodies specific to cows’ milk proteins such as β-lactoglobulin (BLG) or caseins, the major cows’ milk allergens [1,2]. Recent studies suggest that high consumption of peanut in the household during infancy is a risk factor for peanut allergy. Lack and collaborators suggested that cutaneous exposure to low-doses of a food would induce sensitisation while early consumption of food would rather induce oral tolerance [3]. It is possible for humans to be exposed to milk proteins *via* the skin as it enters into the composition of many products applied to the skin. As an example, anaphylaxis in a 12-month-old boy has been described after skin application of an ointment containing milk protein such as caseins [4]. A study also demonstrates the presence of BLG in house dust suggesting that BLG could be available for skin contact and/or inhalation [5].

Most animal models studying epicutaneous administration demonstrated that disruption and inflammation of the skin by tape stripping is necessary to induce efficient sensitisation [6-9]. One study also described efficient cutaneous sensitisation to milk proteins without skin abrasion [10]. However, in our opinion, this model didn’t reflect realistic environmental exposure as the protein applications were performed using high doses of proteins and a patch applied for 24 hours, which may increase the skin permeability and therefore favour penetration of allergens. It has also been shown that allergic sensitisation to α-lactalbumin can be induced by intra-nasal exposure in mice but needs the use of adjuvant [11]. However, to our knowledge, the effect of short time exposures to cow’s milk through an intact skin or intra-nasally, at the same time as the impact of such administration on further oral exposure has not been extensively investigated. We recently demonstrated in mice that cutaneous or respiratory exposures to peanut proteins through intact epithelia induce a Th2 priming. Moreover, this pre-exposure favours subsequent oral sensitization without the need of Th2 mucosal adjuvant [12]. We thus aimed to extend our observations to cows’ milk, a major allergenic food in infants by assessing the immune impact of repeated short-term cutaneous applications on intact skin or of repeated respiratory administrations of allergens from cow’s milk in BALB/c mice.

## Materials and methods

Commercial unheated microfiltrated cows’ milk (Lait Marguerite, Villefranche-sur-saône, France) was used as whole milk (thereafter called “milk”). BLG was purified from the same source of milk using selective precipitation and chromatography and characterized as previously described [1,13]. BLG concentration in cows’ milk was confirmed using specific BLG immunoassay, ensuring that mice receiving pure BLG or milk received the same quantity of BLG [14].

Female BALB/cJ mice, 5 week-old, were purchased from CERJ (Centre d’Elevage René Janvier, Le Genest-Saint-Isle, France), and were housed in filtered cages under normal Specified Pathogen Free husbandry conditions (autoclaved bedding and sterile water). Mice were acclimated for 2 weeks before experimentation. They received a diet in which BLG was not detected using specific immunoassays developed in the laboratory [14]. All animal experiments were performed according to European Community rules of animal care and with authorization N° 91–368 of the French Veterinary Services.

**Figure 1:**
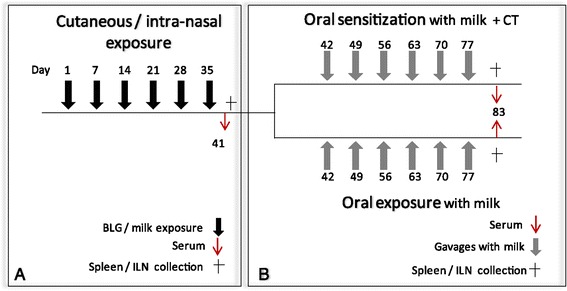
**Experimental schedule [** 12 **]. (A)** In a first set of experiments, mice were exposed to proteins on intact skin. Mice were anaesthetized and their abdomens were carefully shaved with an electric clipper. The remaining hairs were removed using a depilatory cream (Veet®) and the skin was then gently cleaned with water. Skin treatments were performed 4 to 6 days after depilation to be sure that no more lesions or inflammation are present [12]. Once a week for 6 weeks, 100 μg of purified BLG diluted in 30 μl of DPBS or 30 μl of milk containing the equivalent amount of BLG was then spread on the depilated skin of anaesthetized mice (n = 19-20/group). After 40 minutes, skin was gently cleaned with water. Control mice received 30 μl of PBS (n = 19/group). According to the same schedule, other groups of mice were exposed *via* the respiratory tract by intranasal administration of 50 μg of purified BLG diluted in 10 μl of DPBS or 10 μl of milk containing the equivalent amount of BLG (n = 20/group). Control mice received 10 μl of PBS. In both the experimental groups, after last administration, 6 mice per group were sacrificed. Spleen and ILN were removed and cells isolated. Systemic specific cytokine secretion was then assessed after *ex vivo* restimulation with 20 μg of BLG. **(B)** After the six cutaneous or intra-nasal exposures, the remaining mice from each of the experimental groups were separated into 2 subgroups (n = 7-8/subgroup). One group was experimentally sensitized by gavage with 6 mg of milk proteins mixed with *Cholera toxin* (10 μg/administration) [18]. The other mice received 6 mg of milk proteins in the absence of CT. Control mice consisted of mice pre-exposed with PBS then gavaged with milk +/− CT. Some mice were not exposed at all (naive mice; n = 10).

In a first set of experiments, we compared the effect of cutaneous or respiratory exposure to purified BLG or cows’ milk on the immune response to BLG as previously described (Figure 1A) [12]. Mice were then further either orally exposed to cow’s milk or experimentally sensitized to cows’ milk via the oral route using Cholera toxin (CT) as an adjuvant (Figure 1B). The humoral response was assessed by quantitative measurements of BLG-specific IgE and IgG1 antibodies on individual serum samples collected at the end of the two stages of the protocol as described elsewhere [15,16]. The cellular response was assayed after restimulations of immune cells from spleen collected at the end of the two stages of the protocol. Organs were pooled within groups to assess cytokine production under specific *ex vivo* re-stimulation, as described previously [17]. Specific secretion was calculated by subtraction of non-specific secretion induced by ovalbumin. No statistical analysis was performed as results were obtained from pooled organs.

Data were not normally distributed thus a non-parametric test was performed, using the Kruskal-Wallis test followed by Dunn’s multiple comparisons test (DMCT). Statistical analyses were performed using GraphPad Prism 5.01 software (GraphPad software, San Diego, CA, USA).

## Results

**Figure 2:**
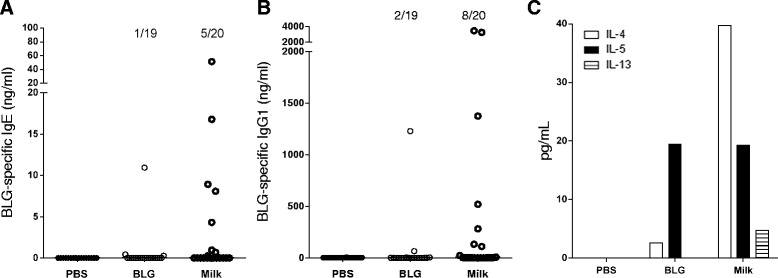
**Immune response induced after cutaneous applications of BLG or whole milk.** Mice received 30 μl of PBS, purified BLG or milk (n = 19-20/group) on intact skin. Serum samples were collected after 6 cutaneous applications and BLG-specific IgE **(A)** and IgG1 **(B)** antibodies were quantified on individual serum samples. The numbers of positive mice are indicated above each group. Results are reported in ng/ml derived from standard curves obtained with calibrated anti-BLG purified monoclonal IgG1 or IgE antibodies [15]. Non-specific binding (NSB) was determined using sera from naive mice. A response was considered significant when higher than NSB + 3σ. **(C)** Systemic specific IL-4, IL-5 and IL-13 secretions were assessed after *ex vivo* restimulation of splenocytes with BLG.

After 6 cutaneous applications, milk triggered significant BLG-specific IgE and IgG1 responses in 5 and 8 out of 20 mice, respectively (Figure 2A and B). Conversely, only 1 and 2 mice out of 19 showed BLG-specific IgE and IgG1, respectively, in the group that received the purified BLG. Systemic BLG-specific Th2 cytokine secretion was also detected in both groups of mice exposed to BLG or milk (Figure 2C). We also detected low systemic IFN-γ secretion in cells from BLG or milk treated mice (data not shown).

**Figure 3:**
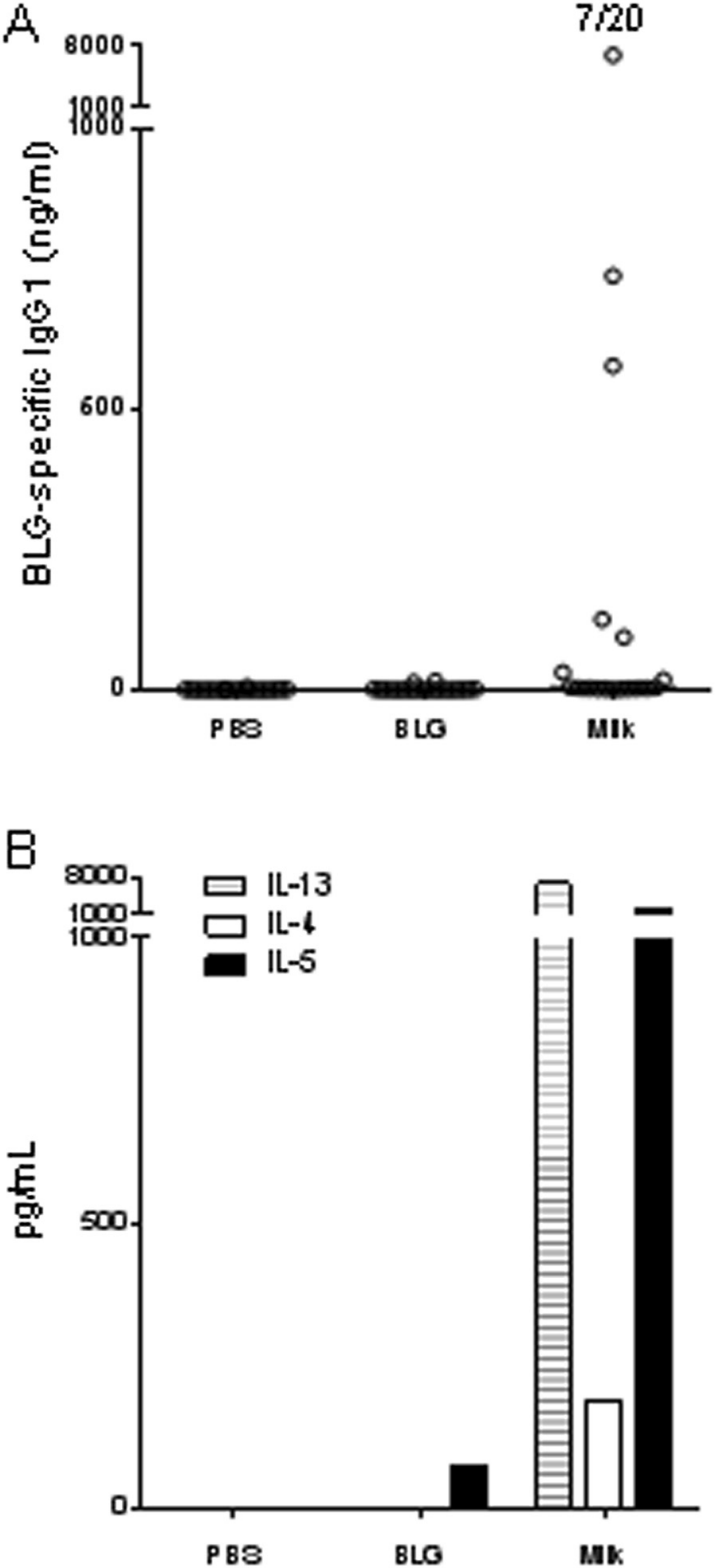
**Immune response induced after intra-nasal administrations of BLG or whole milk.** Mice received 10 μl of PBS, purified BLG or milk intranasally (n = 20/group). **(A)** Serum samples were collected after 6 intra-nasal administrations and BLG-specific IgG1 were quantified on individual serum samples. The numbers of positive mice are indicated above each group. A response was considered significant when higher than NSB + 3σ. **(B)** Systemic specific IL-13, IL-5 and IL-4 secretion was assessed after *ex vivo* restimulation of splenocytes with BLG.

In contrast, no specific IgE could be detected after 6 intra-nasal exposures (not shown) whereas intra-nasal administrations of milk but not of BLG induced BLG-specific IgG1 response in 7 out of 20 mice (Figure 3A). Systemic secretion of IL-13, IL-4 and IL-5 was evidenced in cells from milk-treated mice but not from control or BLG-treated mice (Figure 3B). No BLG-specific IFNγ secretion was detected, irrespective of the group treated.

**Figure 4:**
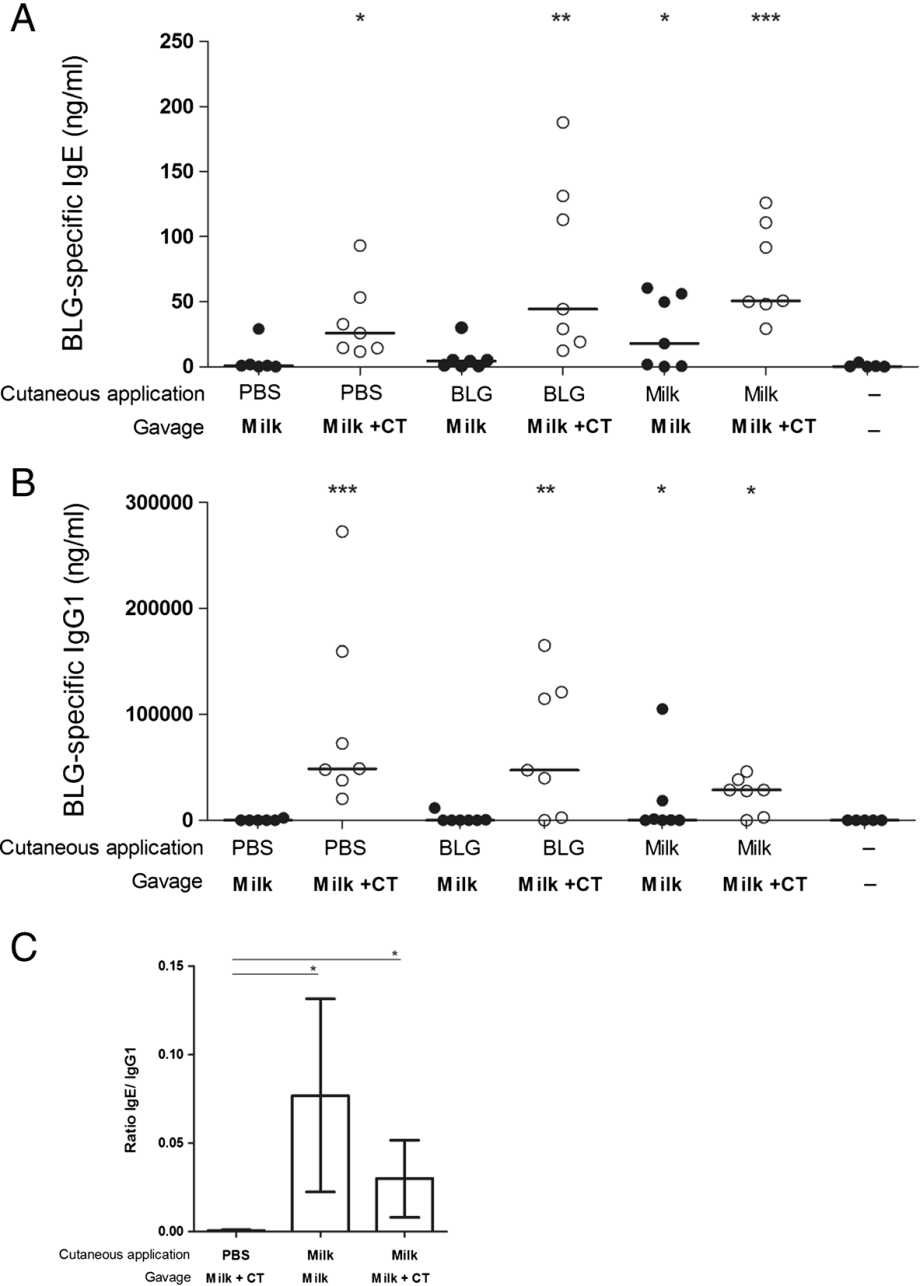
**Impact of cutaneous pre-exposition on further oral exposure to milk +/− CT.** Mice pre-exposed via the cutaneous route to PBS, BLG or milk received 6 mg of milk proteins in the presence (○) or absence (●) of CT (n = 7-8/group) according to the protocol depicted in Figure 1. Serum samples were collected at day 83 and BLG-specific IgE **(A)** and IgG1 **(B)** antibodies were quantified on individual serum samples. *p < 0.05, **p < 0.01, ***p < 0.001 using Kruskal-Wallis and Dunn’s multiple comparison post test when compared to naive group. **(C)** BLG-specific IgE/IgG1 ratio between PBS or milk pre-exposed mice at the end of the protocol.

**Figure 5:**
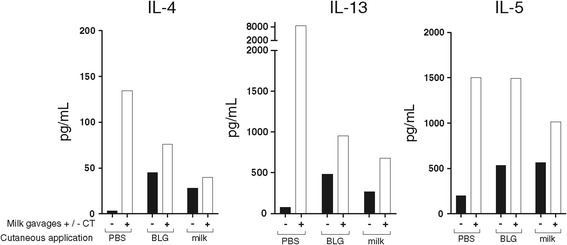
**Cytokine secretion by reactivated splenocytes.** At the end of the protocol, mice from groups pre-exposed by the cutaneous route and further gavaged with milk in the presence (□) or in the absence (■) of CT were sacrificed. Systemic specific cytokine secretion was assessed after *ex vivo* restimulation of splenocytes with 20 μg of BLG. IL-4, IL-13 and IL-5 were assayed using mouse cytokine kit and BioPlex apparatus (Biorad) following manufacturer’s instructions. Specific secretion was calculated by subtraction of non-specific secretion induced by ovalbumin. No statistical analysis was performed as results were obtained from pooled organs.

We further assessed the effect of cutaneous exposure to BLG or to milk on subsequent oral exposure to milk in the presence or absence of the Th2 mucosal adjuvant CT. As expected, we observed that control mice, i.e. pre-exposed to PBS by cutaneous route and then experimentally sensitized by oral exposure to milk in the presence of CT, were efficiently sensitized to BLG as they displayed significant BLG-specific IgE and IgG1 (Figure 4A and B) and high BLG-specific Th2 cytokine secretion (Figure 5) when compared to naive mice. Milk or BLG cutaneous pre-exposition also led to significant sensitisation after the oral administration of milk in the presence of CT, but no difference was evidenced when compared to PBS pre-exposed mice. In the absence of CT, PBS and BLG pre-exposed mice did not produce significant specific IgE or IgG1 at the end of the protocol (Figure 4A and B). No specific Th2 cytokine secretion was detected in corresponding PBS-treated mice, whereas BLG pre-exposure led to significant cytokine production (Figure 5). This suggests the induction of BLG-specific Th2 cells in those latter mice, although no specific IgE could be evidenced. Conversely and as expected from the results shown in Figure 2A, mice cutaneously pre-exposed to milk produced significant BLG-specific IgE (Figure 4A), IgG1 (Figure 4B) and Th2 cytokines (Figure 5) after 6 gavages with milk without CT. Interestingly, the IgE/IgG1 ratio was higher in milk pre-exposed mice when compared to mice pre-exposed to PBS and then experimentally sensitized irrespective of the use of CT (Figure 4C).

**Figure 6:**
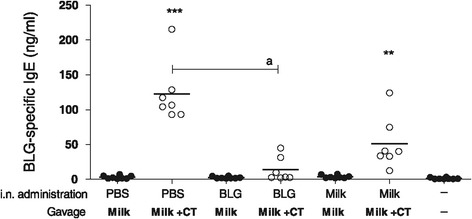
**Impact of intra-nasal pre-exposition on further oral exposure to milk +/− CT.** Respiratory pre-exposed mice (PBS, BLG, milk) received 6 mg of milk proteins in the presence (○) or absence (●) of CT (n = 7-8/group) according to the protocol depicted in Figure 1. Serum samples were collected at day 83 and BLG-specific IgE antibodies were quantified on individual serum samples. *p < 0.05, **p < 0.01, ***p < 0.001 using Kruskal-Wallis and Dunn’s multiple comparison posttest when compared to naive group. a indicates p < 0.05 using Kruskal-Wallis comparison between specified group.

In parallel, we assessed the impact of intranasal pre-exposure on subsequent oral exposure to milk in the presence or absence of Th2 mucosal adjuvant. As expected, PBS pre-exposed mice displayed a significant BLG-specific IgE production after gavages with milk and CT whereas no significant response was evidenced in the absence of CT (Figure 6). Mice that received oral administrations of milk without the adjuvant did not produce any BLG-specific IgE whatever the pre-exposure (Figure 6). Interestingly, mice that were intra-nasally pre-exposed to BLG did not produce significant BLG-specific IgE (Figure 6) after oral gavages with milk in the presence of CT compared to naïve mice. Moreover, the production of IgE in BLG pre-exposed mice was significantly lower from PBS pre-exposed mice. The absence of Th2 cell activation in these mice was confirmed by analysis of cytokine secretion (not shown). Mice pre-exposed to milk then experimentally sensitized demonstrated an intermediate profile: The levels of BLG-specific IgE were slightly lower in milk pre-exposed mice than that observed in PBS pre-exposed mice but the difference was not statistically significant. The same results were observed with BLG-specific IgG1 (not shown).

## Discussion

In the present work, we exposed mice to milk or a purified milk allergen through intact skin [12], by mean of applications not exceeding 40 minutes and without any patch. As respiratory exposure to food proteins can also induce Th2 responses [11,19], we also assessed the effect of intra-nasal administration of milk allergens.

We first demonstrated that cutaneous exposure to milk induced the production of BLG-specific IgE and IgG1 while purified BLG did not. Milk exposure by the intranasal route was also more immunogenic than purified BLG exposure. In milk-treated mice *via* the cutaneous or i.n. route, response was mainly directed against BLG. In both groups, no specific response against lactoferrin was evidenced and only 3 mice out of 20 produced IgG1, but not IgE, against whole caseins (not shown). Systemic casein-specific secretion of IL-5 and IL-13 was detected only after intranasal exposure to milk. These results thus suggest an important role of the milk matrix on BLG presentation to the immune system and/or on its activation. Milk contains several immunomodulatory proteins [20] and fat that could act as an adjuvant. Indeed, a specific lipid fraction of Brazil nuts has been demonstrated to be involved in the immunogenicity of Ber e 1, a brazil nut major allergen [21].

We previously observed that cutaneous or intranasal exposure to purified Ara h 1 induced a strong Ara h 1-specific IgG1 although no IgE could be detected. It is interesting to note that Ara h 1, a large protein (62 KDa) was more immunogenic that a smaller one, i.e. BLG (18 KDa), especially through the skin or pulmonary epithelia. This was also demonstrated after intra-peritoneal administrations of the corresponding purified proteins, then further suggesting different intrinsic allergenic properties of these major allergens [22].

When we further gavaged cutaneously pre-exposed mice without CT, only milk pre-exposed mice produced specific IgE, demonstrating that cutaneous pre-exposure to milk favours further oral sensitisation without the need for a Th2 adjuvant. Although we did not assess the clinical allergy outcome, for instance through an oral challenge with milk or purified BLG, the model presented here, using a first Th2 priming stage of environmental-like exposure then a second stage of oral exposure, could be used as a relevant model of food allergy sensitisation without adjuvant.

Conversely, intra-nasal pre-exposure to milk did not allow subsequent oral sensitisation without the use of the Th2 adjuvant. Intra-nasal pre-exposure to BLG even seems to prevent further sensitization experimentally induced by oral gavages with milk in the presence of CT, as corresponding mice did not produced specific IgE nor Th2 cytokines after reactivation of spleen cells (Figure 2). Intra-nasal pretreatment with BLG did not lead to significant Th2 nor Th1 response, both at the cellular and humoral levels, suggesting the absence of induction of effector specific and/or the induction of immune tolerance. A high dose of BLG administered *via* the gastrointestinal tract also induces efficient tolerance with induction of Tregs [17]. Indeed, Tregs can be promoted *via* an allergen exposure through respiratory route [23] and in steady state conditions DCs seems to induce Tregs rather than T effectors cells [24]. Further studies aiming at analyzing Treg induction in lymph nodes after intranasal BLG administration could then be interesting, but we can already hypothesize that the oral route is not the only one to promote specific tolerance. However, the balance between tolerance and sensitisation appears to be allergen-dependent as we previously demonstrated that respiratory pre-exposure to purified Ara h 1 rather favours subsequent oral sensitisation.

In summary, these results further suggest that non gastrointestinal exposure to food proteins, i.e. via the cutaneous or respiratory route, may lead to Th2 priming facilitating a subsequent sensitization via the oral route. However, this priming to food proteins is dependent on the allergen considered, the route of exposure and the matrix containing the allergen. Moreover, we showed that cutaneous exposure to milk on intact skin favours subsequent oral sensitisation without the use of CT, providing an interesting adjuvant-free experimental model of cows’ milk food allergy combining environmental and oral exposure.

## Abbreviations

BLG: Bovine β-lactoglobulin
CT: *Cholera toxin*
